# Evaluation of coseismic landslide susceptibility by combining Newmark model and XGBoost algorithm

**DOI:** 10.1371/journal.pone.0328705

**Published:** 2025-08-11

**Authors:** Cong Zhang, Zifa Wang, Jintao Xiao, Zhaodong Wang, Dengke Zhao, Zhaoyan Li

**Affiliations:** 1 School of Civil and Architecture Engineering, Henan University, Kaifeng, China; 2 Institute of Engineering Mechanics, China Earthquake Administration, Harbin, China; 3 Key Laboratory of Earthquake Engineering and Engineering Vibration, Institute of Engineering Mechanics, China Earthquake Administration, Harbin, China; 4 Key Laboratory of Earthquake Disaster Mitigation, Ministry of Emergency Management, Harbin, China; China Construction Fourth Engineering Division Corp. Ltd, CHINA

## Abstract

Coseismic landslides are among the most perilous geological disasters in hilly places after earthquakes. Precise assessment of coseismic landslide susceptibility is crucial for forecasting the effects of landslides and alleviating subsequent tragedies. This research formulates a comprehensive landslide hazard assessment model by integrating the Newmark physical model with machine learning techniques. The Jiuzhaigou region serves as the study area, utilizing the comprehensive indicator $F_{s}$ from the Newmark model as a principal feature parameter, which integrates several influencing aspects, including rock strata, moisture content, and slope gradient. Monte Carlo simulations (MCS) mitigate uncertainty in geotechnical parameters, augmenting Newmark model findings’ reliability. The sampling approach utilizes Newmark model outcomes to identify nonlandslide locations for developing six landslide susceptibility models, namely N_XGB, N_RF, Dn_XGB, Dn_RF, and independent XGBoost and RF models. Comparative investigation reveals that the hybrid models N_XGB and N_RF, which amalgamate Newmark model outputs with machine learning methodologies, have enhanced performance. The N_XGB model attains superior accuracy, exhibiting an AUC of 0.96 in the Jiuzhaigou region, markedly surpassing alternative hybrid machine learning models and physical models, moreover, $F_{s}$ as a feature exhibits superior predictive accuracy relative to utilizing Newmark displacement. Validation in the Ludian and Luding region results in model accuracies of 0.88 and 0.86, respectively, indicating robust generalizability across various seismic situations. These findings highlight the benefits of integrating physical principles with data-driven methodologies, providing a comprehensive regional landslide risk assessment and management framework.

## Introduction

Earthquakes and their secondary effects frequently result in substantial casualties and economic losses [1]. Since the 20th century, more than 800 earthquakes with magnitudes of 6.0 or greater have occurred in China, resulting in significant damage [2]. The 2014 Ludian Ms6.5 earthquake triggered 1,024 landslides, formed barrier lakes, and impacted 1.0884 million people, causing the collapse of 80,900 houses [3]. Similarly, the 2017 Jiuzhaigou Ms7.0 earthquake triggered approximately 4,834 landslides, leading to 25 fatalities, 6 missing persons, and widespread property damage [4]. Between 2019 and 2023, landslides accounted for 59.33% of all geological disasters nationwide, with seismic landslides being especially severe. These landslides frequently result in rapid, large-scale movement, potentially blocking rivers and forming barrier lakes that exacerbate flooding risk. Therefore, studying seismic landslides is essential for understanding earthquake-induced secondary effects, with research on spatial evolution and hazard distribution playing a key role in post-disaster reconstruction and mitigation [1].

Several methods have been employed for postseismic landslide hazard assessment, including the analytic hierarchy process (AHP) [5], weight analysis [6], Newmark displacement method [7], and machine learning techniques [8–10], The latest review by Shao and Xu [11] indicates that over half (51%) of LASA-related studies focus on these techniques, highlighting their effectiveness in enhancing prediction accuracy. The Newmark displacement ($D_{n}$) method is widely utilized because of its solid theoretical foundation, ease of calculation, and minimal parameter requirements, particularly in regions with limited data. Ma and Xu [12] applied it to assess slope stability following the 2013 Lushan earthquake, and Wang et al. [13] developed a rapid assessment tool for seismic landslides in the Wenchuan region. Similarly, Shinoda et al. [14] optimized geotechnical strength parameters through inversion analysis using the Newmark sliding block model to evaluate regional landslide susceptibility triggered by the Kumamoto earthquake sequence. However, their approach exhibited limited transferability across regions due to reliance on a single seismic event catalog for parameter inversion. Despite these applications and advancements, challenges persist with the Newmark method itself. These include: (1) the use of $D_{n}$ to estimate landslide probability without accounting for other environmental factors, such as hydrology and topography. (2) Reliance on expert judgment for parameter assignments, which introduces variability and compromises accuracy.

With the advancements in big data and artificial intelligence, machine learning has increasingly been used for predicting geological disasters [15–19]. Decision tree models are valuable for interpretability, but they lack generalizability [20]. Ensemble models, such as Random Forest and XGBoost, which utilize bagging techniques, demonstrate improved performance [21,22]. For instance, Zhang et al. [23] applied a SHAP-XGBoost framework to analyze landslide susceptibility across several counties, which demonstrated robust predictive capabilities. Recent advances in XGBoost-based surrogate models demonstrate exceptional efficiency in geotechnical reliability analysis. Zhang et al. [24] showed that their framework accelerated unsaturated slope stability assessment under rapid reservoir drawdown by 3–4 orders of magnitude while maintaining accuracy in low-probability failure scenarios. This underscores the potential of machine learning to overcome computational bottlenecks in physically coupled landslide modeling. XGBoost is particularly effective for large datasets, providing faster training and enhanced accuracy [25]. However, challenges remain: the choice of sampling methods greatly influences model accuracy [26], with random or buffer sampling introducing subjectivity. Additionally, Data-driven approaches alone exhibit significant limitations in predicting earthquake-induced landslides, many studies overlook geotechnical characteristics and landslide mechanics. Karakas et al. [27], during their validation of Turkish earthquake landslides, observed a substantial decline in model generalization accuracy (to 76%). They attributed this decline to the neglect of seismic physical mechanisms and dynamic parameters, such as Peak Ground Acceleration (PGA). Similarly, studies by Matsakou et al. [28] (based on the Lefkada Island earthquake) and Umar et al. [29] (2014 West Sumatra earthquake) relied solely on static environmental factors (geology, topography), overlooking critical seismic triggering factors or hydrological conditions. This omission constrains the physical interpretability and predictive capability of these models for specific seismic events. Although machine learning methods, such as Random Forest and Multilayer Perceptron, have shown potential in post-event validation (Karakas et al. [30], 2022, for the Elazig earthquake), their black-box nature and neglect of geomechanical principles hinder the explicit integration of landslide physical mechanisms. Unlike the Newmark model, machine learning relies on extensive labeled data, which may suffer from data imbalance, thereby affecting outcomes.

To enhance the predictive accuracy and generalizability of landslide susceptibility assessments, this study proposes a hybrid approach that integrates the Newmark physical model with data-driven machine learning techniques, addressing both parameter uncertainty and limitations in feature selection. Unlike previous studies that solely employed Newmark displacement $D_{n}$ as a discrete risk label, the computed static factor of safety $F_{s}$ is incorporated as a continuous numerical feature in the data-driven model. $F_{s}$ captures the static resistance and potential instability of slopes prior to landslide initiation, thereby reducing information loss and allowing a more precise characterization of slope stability. To address the inherent uncertainty of geotechnical parameters (cohesion, friction angle, unit weight), the study categorizes the region by engineering rock type. It applies Monte Carlo simulation (MCS) within each group to generate realistic parameter distributions and reduce uncertainty.

Regarding adverse sample selection, traditional methods often employ buffered or random sampling, which may introduce spatial bias and increase false-positive rates. In contrast, this study extracts non-landslide points exclusively from “very low” and “low” risk zones identified via cumulative Newmark displacement values, maintaining a 500 m buffer from known landslide areas. This physically informed sampling strategy ensures both the physical stability of negative samples and their comparability with landslide samples in terms of terrain and geological characteristics.

The feature set incorporates ten key variables, including topographic factors (slope, aspect, elevation, curvature), geological factors (lithology, fault distance), anthropogenic factors (distance to roads, rivers), and seismic factors (epicentral distance, peak ground acceleration), to comprehensively evaluate slope susceptibility under various conditions. Six models were constructed based on both individual and hybrid methods. A comparative analysis of $F_{s}$ and $D_{n}$ within different frameworks was conducted to identify the most reliable physical indicator for landslide susceptibility prediction and interpretation.

## Area and data

### Study and validation area

**Fig 1:**
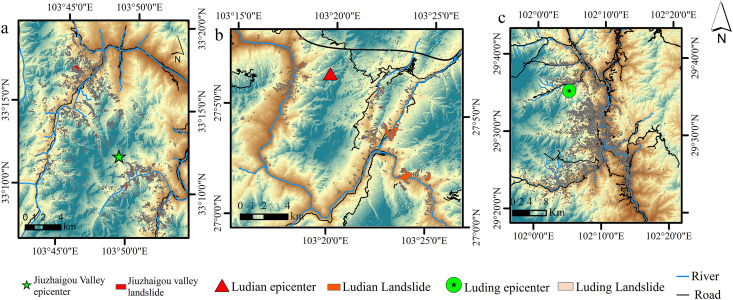
Geographic overview and locations of landslides. **(a)** Jiuzhaigou study area; **(b)** Ludian validation area; **(c)** Luding validation area. Source: The boundaries, roads, and rivers used in the map are derived from http://www.naturalearthdata.com/. The map was originally produced by the authors.

Jiuzhaigou County, located in the northeastern part of Sichuan Province, and Ludian County, northeast Yunnan Province, are seismically active regions characterized by complex mountainous terrain and significant geological structures. Jiuzhaigou lies within a transitional zone between the Qinghai-Tibet Plateau and the Sichuan Basin. Ludian is part of the southeastern Hengduan Mountains, influenced by the active Ludian-Qiaojia Fault Zone. Luding, located in western Sichuan, features typical plateau-mountain terrain and is traversed by the seismogenic Xianshuihe Fault Zone. Its deeply incised river valleys (e.g., Dadu and Yalong tributaries) and fractured bedrock create high susceptibility to coseismic landslides [31]. The 2014 Ludian Mw6.5, 2017 Jiuzhaigou Mw7.0, and 2022 Luding Mw 6.6 earthquakes triggered widespread landslides. The Jiuzhaigou earthquake caused 25 deaths, 525 injuries, and damage to more than 73,000 houses [32], whereas the Ludian earthquake led to numerous landslides near the epicenter [3,33]. The Luding earthquake (epicenter: 102.08°E, 30.33°N; depth: 16 km) generated dense landslides along steep valleys, exemplified by the Moxi Town collapse. Fig 1 provides a geographic overview of regional landslides on the DEM maps of the studied area.

### Data preprocessing

Landslide data were obtained from the United States Geological Survey (USGS) and the National Glacial, Permafrost, and Desert Data Center (http://www.ncdc.ac.cn). Additional data were sourced from official websites, including the National Geological Archives, the National Earth System Science Data Center (https://www.geodata.cn), the National Seismic Science Data Center, and the Resource and Environment Science Data Center of the Chinese Academy of Sciences. Specific data sources are detailed in Table 1.

**Table 1:** Description and sources of datasets.

Data	Data sources	Style	Accurate	Purpose
Jiuzhaigou Valley landslide	National Glacial Tundra Desert Science Data Center	Vector	——	Actual landslide data
Ludian landslide	the United States Geological Survey	Vector	——	Actual landslide data
Luding landslide	the United States Geological Survey	Vector	——	Actual landslide data
DEM	Geospatial data cloud	Raster	30 meters	Extracting factors such as slope direction, slope gradient, plane curvature, etc.
Geological data	National Geological Information Data Center	Vector	1:2000000	Extracting formation information
PGA data	the United States Geological Survey	Vector	——	PGA ground shaking parameters in the study area
Road data	Data Center for Resource and Environmental Sciences, Chinese Academy of Sciences	Vector	1:1000000	Extraction study regional roads
Hydrological data	National Earth System Science Data Center	Vector	1:1000000	Extraction of study area rivers
geologic fault data	National Center for Earthquake Science Data	Vector	——	Extraction of fault data from the study area

**Fig 2:**
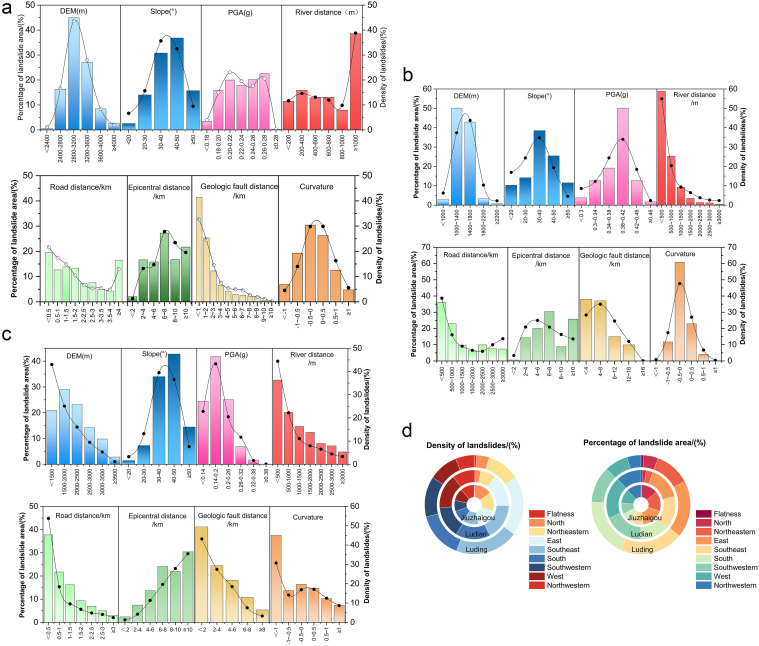
Statistical analysis of factors affecting landslides.

Fig 2a–2c illustrate the data analysis of the landslip features in Jiuzhaigou, Ludian and Luding, while Fig 2d presents the study of slope direction. The Jiuzhaigou earthquake triggered 4,834 landslides, the majority of landslides have an area ranging from 100 to 400 m^2^ and are predominantly classified as small- to medium-sized events. A total of 596 landslides have an area greater than 2,000 m^2^, among which only two exceed 50,000 m^2^, with areas of 55,200 m^2^ and 236,300 m^2^, respectively. These two large landslides are both located near the central fault zone. The total landslide area amounts to 9.55 km^2^, 71% at 2,400–3,200 m elevations and 67% on slopes of 30° to 50°. Most events occurred on northern, northeastern, and eastern slopes, likely due to southeastward seismic wave propagation. Landslide susceptibility increased with increasing PGA but decreased beyond 0.28 g. Few landslides occurred near the epicenter, and most were more than 10 kilometers from the fault, over 1 kilometer from rivers, and near roads.

The Ludian earthquake triggered 1,024 landslides, the overall landslide distribution exhibits a northwest-southeast reverse pattern. A total of 1,024 coseismic landslides covered a cumulative area of 5.19 km^2^. The largest landslide covered 0.406 km^2^, while the smallest was 101 m^2^. Among them, 473 landslides with areas greater than 2,000 m^2^ accounted for 46.2% of the total, while 94 landslides more minor than 500 m^2^ accounted for only 9.2%. Although the Ludian earthquake affected a relatively small area, it produced a larger number of landslides, many of which were large in scale and volume, mostly between 1,400 and 1,800 m elevations, peaking on slopes of 30° to 40°. Landslides were concentrated on eastern, southeastern, and southern slopes, with a higher frequency within 6–8 kilometers from the epicenter. Most landslides occurred at PGA values between 0.38g and 0.42g, particularly near roads and rivers and within 4–8 km of the fault.

In the Luding area, a total of 10,855 earthquake-induced landslides were recorded, covering a cumulative area of 47.3 km^2^. Of these, 4,600 landslides (42.4%) exceeded 2,000 m^2^, 2,721 landslides (25%) were smaller than 500 m^2^, and the remaining 32.6% were of moderate size. Landslides were predominantly concentrated at elevations of 1,300–1,700 m (68%) and on slopes of 25°–35° (62%), which are lower and gentler than those in Jiuzhaigou and Ludian. The aspect was mainly east to southeast (56%), contrasting with north–northeast aspects in Jiuzhaigou and east–southeast aspects in Ludian. Landslides most frequently occurred under PGA values of 0.36–0.44 g (74%), exceeding the trigger thresholds observed in Jiuzhaigou. Forty-two percent of landslides were located 6–10 km from the epicenter, and 48% were 4–6 km from active faults. Landslides occurring within 800 m of rivers and 600 m of roads accounted for 55% and 50%, respectively, significantly higher than in Jiuzhaigou and Ludian.

A three-region comparison reveals that Jiuzhaigou is dominated by small-to medium-sized landslides at higher elevations; Ludian exhibits a moderate number of landslides spanning a wide size range; and Luding is characterized by predominantly medium-to large-sized landslides occurring at mid-elevations within fault-controlled zones, influenced by both high PGA and hydrological–roadway factors. These differences reflect significant variations in topography, lithology, and seismic conditions among the regions, providing a solid geological context for subsequent cross-regional model generalization and validation.

## Methods

### The Newmark physical model

The model simulates landslides as rigid body movements along a slope, where permanent displacement results from seismic loading. It assesses slope stability by calculating the cumulative displacement [34].

The critical acceleration $a_{c}$ is derived from equilibrium theory:


$$\begin{array}{c} a_{c}=\left(F_{s}-1\right)g\sin\alpha \; \end{array}$$ (1)

where g is the gravitational acceleration and where α is the slope angle. The static safety factor $F_{s}$ is calculated by:


$$\begin{array}{c} F_{S}=\frac{c^{\prime }}{\gamma t\sin\alpha }+\frac{\tan\varphi^{\prime }}{\tan\alpha }-\frac{m\gamma_{w}\tan\varphi^{\prime }}{\gamma \tan\alpha } \end{array}$$ (2)

Here, $c^{\prime }$ is effective cohesion, $\varphi^{\prime }$ is the effective friction angle, γ is unit weight, t is sliding plane thickness, and m is the saturation degree [12,35].

Owing to limited seismic data, an empirical Newmark fitting formula incorporating seismic motion and critical acceleration is commonly applied. The equation proposed by Jibson [36], which uses the ratio of ac to amax, representing the peak horizontal ground acceleration (PGA) recorded or estimated at the ground surface during an earthquake, provides the best fit and is adopted in this study.


$$\begin{array}{c} \log D_{n1}=0.215+\log\left[{\left(1-\frac{a_{c}}{a_{max}}\right)}^{2.341}{\left(\frac{a_{c}}{a_{max}}\right)}^{-1.438}\right] \end{array}$$ (3)

$$\begin{array}{c} \log D_{n2}=-2.71+\log\left[{\left(1-\frac{a_{c}}{a_{max}}\right)}^{2.335}{\left(\frac{a_{c}}{a_{max}}\right)}^{-1.478}\right]+0.424M_{w} \end{array}$$ (4)

### XGBoost

The XGBoost algorithm, developed by Chen and Guestrin [37] represents an advanced enhancement of the gradient-boosting decision tree (GBDT) method. Multiple decision trees are combined to form a single strong learner by aggregating their predictions. This is achieved by introducing a “bias” term into the objective function and incorporating regularization to mitigate overfitting, reduce tree complexity, and enhance performance.

### RF

The random forest (RF) algorithm, introduced by Breiman [38], is a bagging-based ensemble learning method that works by creating multiple decision trees. The final prediction is achieved by averaging the prediction values from each tree. Increasing the number of trees in the forest helps in convergence without causing overfitting, thus reducing the generalization error [39,40].

**Fig 3:**
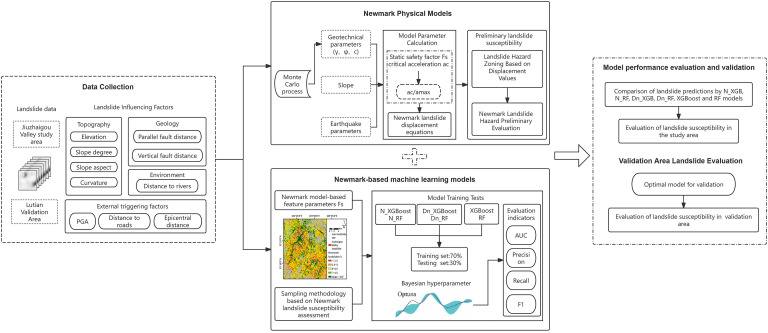
Research technology roadmap.

Fig 3 presents a detailed representation of the hybrid architecture employed for seismic landslip susceptibility evaluation, including the complete process from data acquisition and deterministic modeling to the final predictive analysis.

## Results and analysis

### Newmark-based physical parameters of the formation

Precisely predicting rock layer parameters is crucial for determining $F_{s}$ in the Newmark model [41]. Conventional techniques, dependent on extensive sampling and laboratory analysis, are laborious and expensive, particularly for large-scale hazard evaluations [42]. Geological maps are frequently employed for lithological classification, utilizing parameters computed according to industry standards [12]. Nonetheless, these methodologies fail to address the variety and uncertainty intrinsic to actual geotechnical conditions [43].

**Fig 4:**
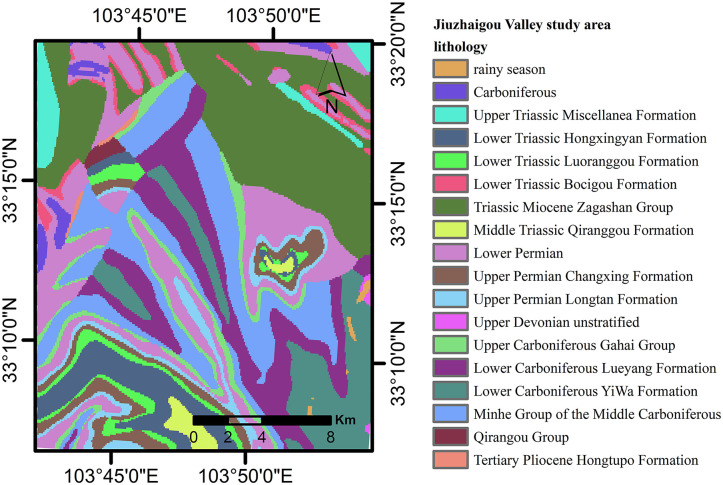
Rock formations in the study area.

This research effectively utilized Monte Carlo simulations (MCS) to overcome the constraints of conventional techniques and to measure uncertainty and variability in geotechnical parameters. The study region was categorized into three engineering rock types—complex, moderately challenging, and soft—utilizing 1:200,000 geological data based on lithology, weathering, and genesis (Fig 4). Hard rock consisted of dense dolomite and slate from the Upper Triassic, whilst moderately hard rock included limestone, bioclastic limestone, and dolomitic limestone from the Carboniferous, Triassic, and Permian periods. Bioclastic limestone with muddy interlayers and dolomitic rocks, including mudstone from the Lower Triassic, were recognized as more susceptible to weathering.

**Fig 5:**
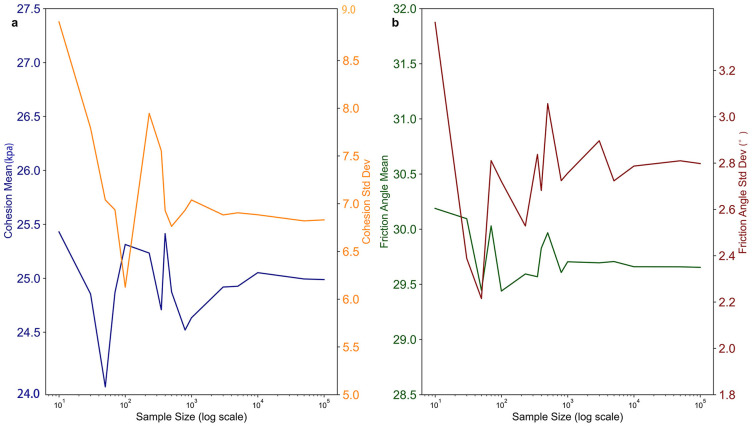
Stabilization of key parameters with increasing sample size for Softer Rock Group. **a** Effective cohesion (∁), **b** Effective friction angle (φ).

The essential geotechnical parameter-effective friction angle (φ), effective cohesiveness (C), and unit weight (γ)—for standard rock types are consolidated in Table 2. The effective friction angle and cohesion were represented using Gaussian distributions, with the mean and standard deviation values specified in the table. Monte Carlo simulations produced 10,000 random samples for each lithological unit. The sample size was established by monitoring the stabilization of statistical measures, including mean and variance, with the rise in sample size. Fig 5 depicts several factors’ outcomes for a particular lithological unit (Softer rock group). The investigation indicated that with 10,000 samples, statistical metrics converged adequately, with negligible accuracy improvements from additional sample size increments.

**Table 2:** Values of physical parameters of engineered rock formations.

Grouping of rock strata	c/kpa		φ/(∘)		γ/(kN/m³)
	Mean	Standard deviation	Mean	Standard deviation	
Hard rock group	35	10	40	4	25
Harder rock group	30	9	35	3	23
Softer rock group	25	7	30	3	20

**Fig 6:**
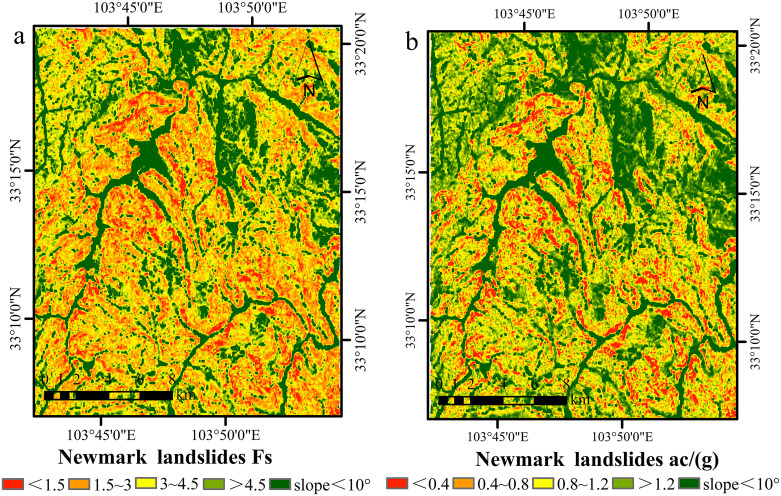
Map showing distribution of static factor of safety and critical acceleration. **(a) **Static factor of safety $\begin{array}{c} F_{s} \end{array}$; **(b)** Critical acceleration $\begin{array}{c} a_{c} \end{array}$. Source: $F_{s}$ and $a_{c}$ were modeled; slopes and regional boundaries were sourced from http://www.naturalearthdata.com/.The map was originally produced by the authors.

After grouping, the parameters of the rock layers and the corresponding slope data were used in Python to calculate the factor of safety ($F_{s}$) for the study area. On the basis of previous statistical analysis and empirical studies, slopes with gradients less than 10° are highly stable and unlikely to experience landslides; therefore, regions with slopes less than 10° were excluded from the calculations [44]. The final calculated $F_{s}$ and $a_{c}$ are shown in Fig 6.

The critical acceleration ($a_{c}$) is closely related to the geotechnical properties of the slope and its gradient. A smaller ac value indicates that the slope is more likely to lose stability under seismic forces, whereas a larger $a_{c}$ value suggests that a higher peak ground acceleration is necessary to trigger slope failure, indicating greater slope stability.

Relying solely on the critical acceleration ($a_{c}$) for stability assessment has limitations, as it emphasizes inherent slope stability while overlooking the varying impacts of seismic motion across different regions. This one-dimensional approach may result in incomplete evaluations [45]. To assess slope performance more accurately during earthquakes, calculating the cumulative sliding displacement ($D_{n}$) is essential, as it accounts for both seismic intensity and slope characteristics, thus providing a more comprehensive evaluation of stability.

### Results of the Newmark landslide hazard assessment

**Fig 7:**
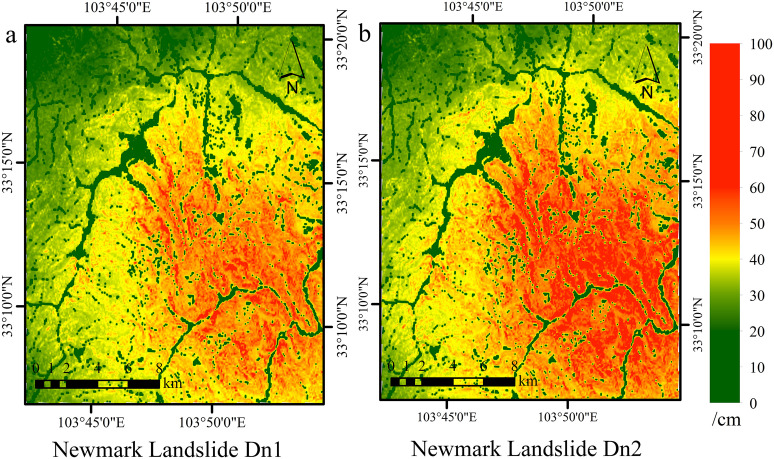
Distribution of Newmark displacement values in Jiuzhaigou valley. **(a)** Dn1; **(b)** Dn2. Source: Dn was modeled; boundaries from http://www.naturalearthdata.com/. The map was originally produced by the authors.

After determining the factors of safety ($F_{s}$) and critical acceleration ($a_{c}$) for the study area, we utilized two displacement prediction equations. The displacement Dn1 was estimated via a single parameter (PGA), whereas Dn2 was calculated using multiple parameters (Mw, PGA). We subsequently developed a Newmark displacement calculation module using ArcGIS spatial modeling functions. This module was then employed to calculate the cumulative landslide displacement distribution in Jiuzhaigou, as depicted in Fig 7.

**Fig 8:**
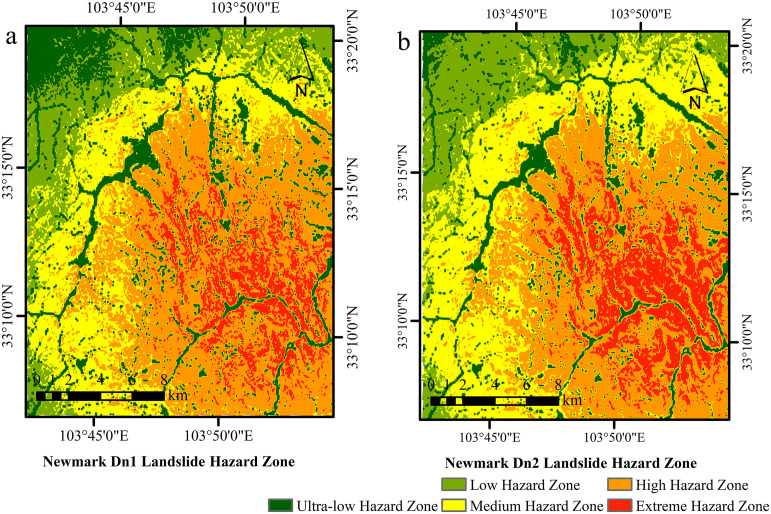
Newmark-based landslide hazard classification. **(a)** Dn1; **(b)** Dn2. Source: Hazard zones modeled; boundaries from http://www.naturalearthdata.com/. The map was originally produced by the authors.

The study assessed seismic landslide hazards based on Newmark displacement results, categorizing the area into five hazard levels (extremely high, high, moderate, low and very low) using a natural break classification. Hazard zones were evaluated, and landslide occurrence within each zone was statistically analyzed in ArcGIS, as illustrated for the Jiuzhaigou region in Fig 8.

In order to compare model performance for different hazard zones, we calculated landslide density (number of occurrences/square kilometer) by dividing the number of correctly predicted landslides by the area of the corresponding hazard zone. Formally, Density of landslides = N/ A, where N is the number of predicted landslides in the area, and A is the area of the area in square kilometers. This normalized metric facilitates comparisons between areas of different sizes and reflects the ability of the model to focus on predicting high-risk areas.

The effectiveness of the Dn1 (PGA) (Table 3) and Dn2 (PGA, Mw) (Table 4) models in predicting landslides across hazard zones was evaluated. Compared with the Dn1 model (3.4 km^2^), the Dn2 model demonstrated superior performance, predicting a larger landslide area in extremely high-risk zones (4.08 km^2^) and achieving a higher landslide density, thereby indicating enhanced predictive accuracy in high-risk areas. In high-risk zones, the predicted landslide area for Dn2 was 3.39 km^2^, surpassing Dn1 prediction of 2.86 km^2^. In low-risk zones, Dn2 was estimated to predict 0.18 km^2^ with a density of 1.67 occurrences/km^2^, significantly lower than Dn1 values of 1.08 km^2^ and 5.56 occurrences/km^2^, thus reducing the occurrence of false positives. Similarly, in very low-risk zones, the predictions from Dn2 (0.11 km^2^, 1.45 occurrences/km^2^) were lower than those from Dn1 (0.56 km^2^, 5.42 occurrences/km^2^), confirming its superior precision.

**Table 3:** Statistical results of the Newmark displacement equation Dn1 hazard zoning.

Landslide hazard class	Landslide area/km^2^	Area size/km^2^	Graded slippage point/occurrences	Density of landslides/ (occurrences/ km^2^)
Extremely high hazard zone	3.4	52.65	1200	22.79
High hazard zone	2.86	131.29	1581	12.04
Medium hazard zone	1.77	119.85	1131	9.44
Low hazard zone	1.08	97.3	541	5.56
Very low hazard zone	0.56	70.3	381	5.42

**Table 4:** Statistical results of the Newmark displacement equation Dn2 hazard zoning.

Landslide hazard class	Landslide area/ km^2^	Area size/ km^2^	Graded slippage point/occurrences	Density of landslides/ (occurrences/ km^2^)
Extremely high hazard zone	4.08	72.15	1701	23.58
High hazard zone	3.39	142.79	1707	11.95
Medium hazard zone	1.91	127.51	1222	9.58
Low hazard zone	0.18	78.47	131	1.67
Very low hazard zone	0.11	50.47	73	1.45

The multiparameter model (PGA, Mw) consistently produced elevated landslide point densities in all hazard zones, especially in high and extremely high-risk regions. Integrating earthquake magnitude (Mw) enhances identifying high-risk areas, yielding more precise and focused landslide forecasts. This methodology improves risk evaluation by concentrating on smaller, high-risk regions.

The Newmark model has inherent limitations. Although Monte Carlo Simulation (MCS) reduces uncertainty in geotechnical parameters by incorporating variations in cohesion, friction angle, and unit weight, discrepancies exist between model predictions and actual observations. High-risk areas are located mainly on the right side of the study area, while severe landslides occur in the low-displacement areas on the left side. This mismatch may be due to the modeling assumptions, as the model only focuses on rigid-body motions and ignores the dynamic interactions between seismic waves and heterogeneous conditions in the subsurface. In addition, the displacement equation ($D_{n}$) derived from empirical seismic data does not adequately consider the amplification of seismic waves, local geology, and complex topography. Under such intricate conditions, there is still a need to improve the accuracy of the model.

### Machine learning feature parameters

Landslides triggered by earthquakes are affected by topography, lithology, geological structure, vegetation cover, seismic forces, and both internal and external triggers [46].

**Fig 9:**
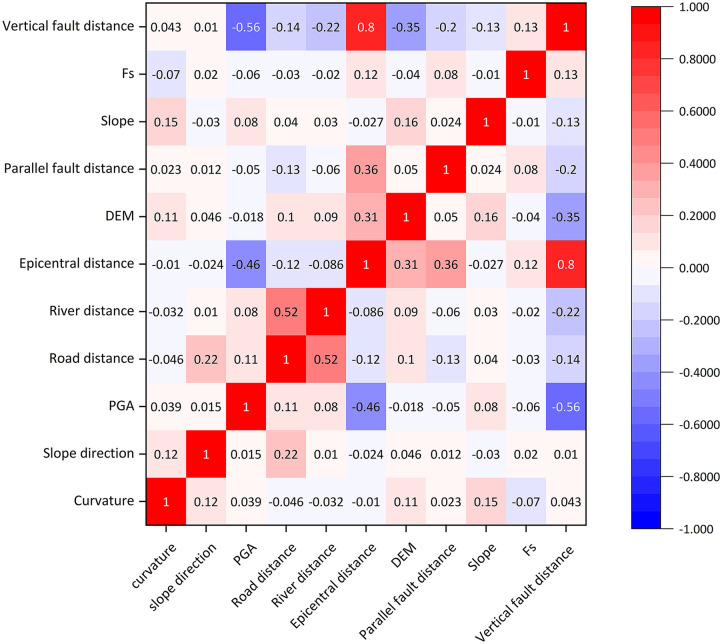
Impact factor pearson correlation coefficient.

**Fig 10:**
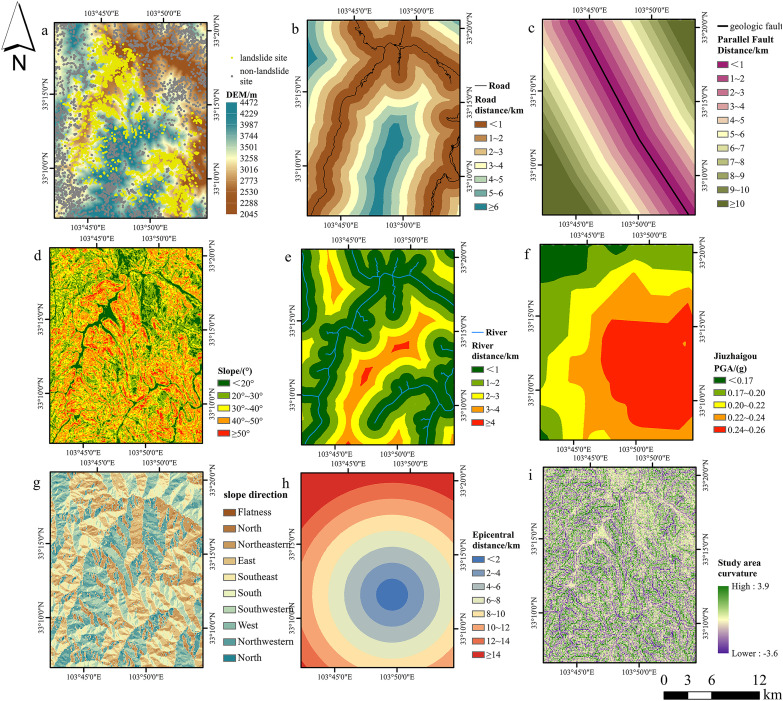
Landslide causal factors. **a** DEM, **b** Distance to road, **c** Distance to parallel fault, **d** Slope, **e** Distance to river, **f** PGA, **g** Slope direction, **h** Distance to epicentral, **i** Curvature. Source: Slope, curvature, slope surface derived from DEM; Roads, rivers from http://www.naturalearthdata.com/; PGA, epicentral distance, faults from USGS. The map was originally produced by the authors.

Physical models often encompass lithological and seismic aspects, although frequently exclude essential external components like highways and rivers. The selection of suitable evaluation parameters is essential for the application of machine learning models in landslide susceptibility evaluations; yet a standardized framework is lacking. Investigations of landslides in hilly valleys categorized data into topography, geology, environmental variables, and external causes. Pearson correlation analysis was employed to investigate 10 parameters from 4,834 landslide samples (Fig 9). A robust correlation (0.8) between epicenter distance and fault distance necessitated the deletion of the latter to prevent multicollinearity, resulting in nine components remaining in the model (Fig 10).

### Physical model-based machine learning feature parameters

The static safety factor is pivotal in landslide stability assessment. According to the Newmark model, it is derived from geological conditions: slope, soil parameters, and seismic factors, including shear strength, unit weight, seismic acceleration, slope angle, soil moisture, friction angle, and cohesion. Consequently, it reflects seismic stability and enhances prediction accuracy when integrated into machine learning models. Traditional machine learning models rely on static geological parameters (layer thickness, lithology, fracture development, groundwater levels, and rainfall) and often neglect dynamic seismic factors, reducing predictive accuracy. The incorporation enables machine learning models to capture dynamic seismic responses better. Studies confirm that integrating from the Newmark model markedly improves machine learning performance in landslide risk assessment over models that use only static geological parameters.

In contrast, the study also explored the incorporation of displacement value (*Dn*) as an alternative physical model feature (Fig 8b). Unlike $F_{s}$, which represents a static safety margin, **D*n* reflects the actual displacement induced by seismic events, providing a dynamic measure of landslide movement. By comparing both $F_{s}$ and **D*n*, the study ensured a robust and dynamic process for landslide prediction.

### Model training

Hyperparameter optimization was conducted for all models, including Random Forest (RF), XGBoost, and their variants (N_XGB, N_RF, Dn_XGB, and Dn_RF). Key hyperparameters were tuned using Bayesian optimization to explore the parameter space efficiently, while noncritical parameters were left at their default settings [47].

The Newmark model divides the study area into five landslide risk levels based on cumulative displacement (extremely high, high, moderate, low, and very low), which guides the selection of samples. Landslide sample points are extracted from interpreted and manually corrected landslide polygons (landslide bodies), resulting in a total of 4,834 earthquake-induced landslide samples. These samples are then randomly divided into a 7:3 ratio, resulting in 3,384 training samples and 1,450 validation samples, to ensure the independence and consistency of the training and validation datasets.

The selection of non-landslide sample points is based on the predictions of the Newmark physical model. First, the landslide displacement of each region is calculated using the Newmark displacement equation, and the natural breaks method is used to divide the area into five risk zones. The two zones with the lowest displacement values are defined as “very low-risk” and “low-risk” areas, which are considered physically stable. Stratified random sampling is conducted within these two low-risk zones. To avoid buffer effects, a 500 m buffer is established around known landslide polygons to ensure that non-landslide points do not overlap with known landslide boundaries. Additionally, stratified random sampling is performed in the “very low-risk” and “low-risk” zones outside the buffer based on the slope and elevation distributions, ensuring the topographical diversity of non-landslide samples. A total of 4,878 non-landslide points are sampled and randomly divided into 3,414 training and 1,464 validation samples using the same 7:3 ratio.

**Fig 11:**
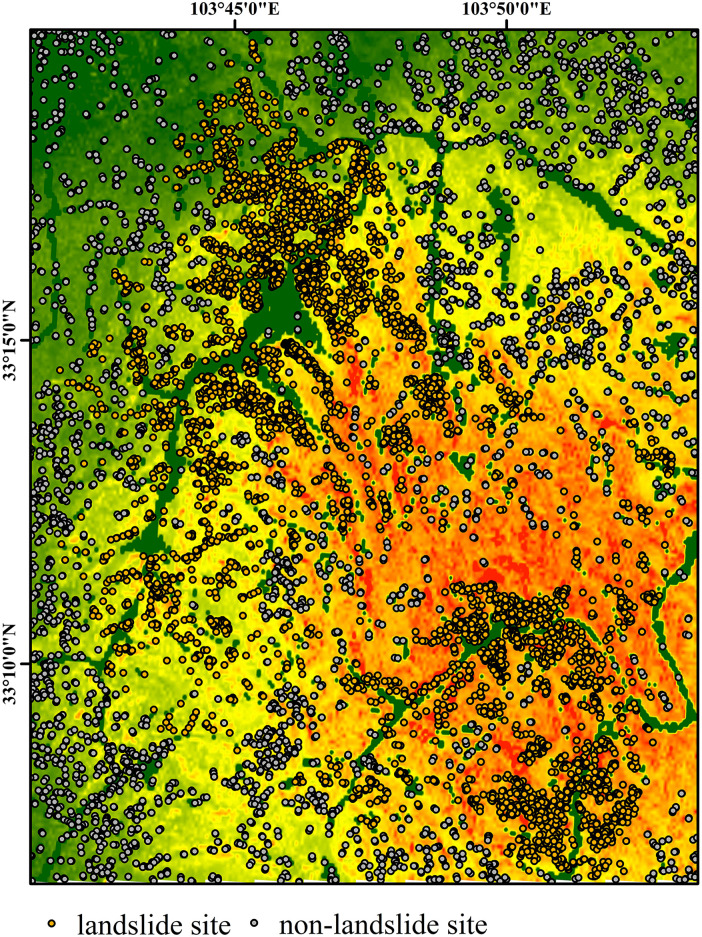
Distribution of landslide sites and non-landslide sites.

This non-landslide sampling strategy, based on low-risk areas identified by the physical model, offers significant advantages. First, it ensures that the selected negative samples are indeed located in physically stable zones as predicted by the Newmark model, thereby minimizing the risk of harmful sample contamination caused by inaccurate displacement thresholds. Second, by sampling within similar slope and elevation ranges as the landslide samples, the topographic comparability between positive and negative samples is enhanced. This enables the model to distinguish between landslide and non-landslide areas more accurately and effectively, improving its generalization capability and robustness. The spatial distributions of both landslide and non-landslide samples are illustrated in Fig 11.

Six models were developed for performance comparisons. N_XGBoost and N_RF integrate the Newmark model into the XGBoost and Random Forest frameworks using the static safety factor as an input feature. Dn_XGBoost and Dn_RF incorporate Newmark-derived permanent displacement ($D_{n}$) as an additional feature. Independent XGBoost and RF models were also tested for comparison but did not include Newmark-derived features. Model performance was assessed using the area under the curve (AUC), mean square error (MSE), root mean square error (RMSE), precision, recall, and F1-score, with AUC denoting prediction accuracy, MSE quantifying the mean squared error, RMSE reflecting prediction error in raw units, whereas precision and recall assess true positive accuracy and completeness. The F1-score provides a balanced metric for overall model evaluation, to provide a comprehensive performance assessment.

### Analysis of hybrid machine learning model landslide assessment results

**Fig 12:**
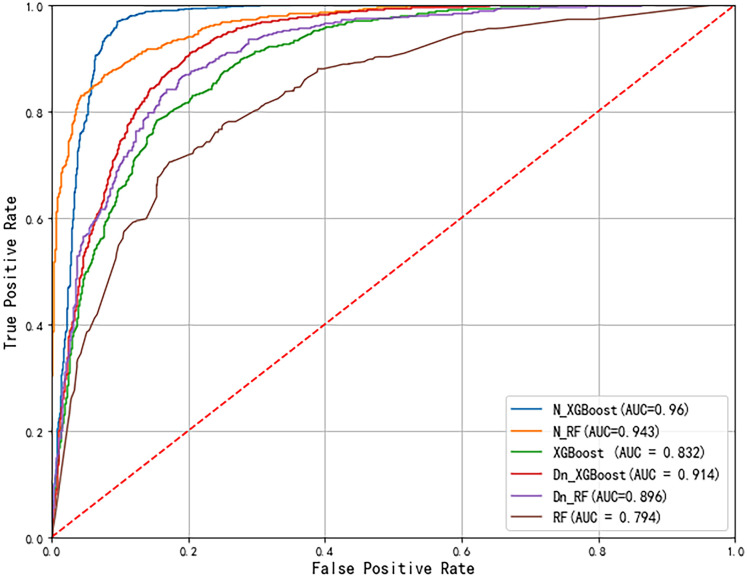
AUC curves for different models.

The results in Fig 12 show that N_XGBoost achieved the highest AUC (0.96), indicating superior predictive accuracy. N_RF was followed by an AUC of 0.943, which demonstrated strong performance owing to the inclusion of static safety factors. Dn_XGBoost and Dn_RF improved upon their standalone counterparts, with AUCs of 0.914 and 0.896, respectively, by utilizing the $D_{n}$ parameter. In contrast, standalone XGBoost and RF models exhibited the lowest performance, with AUCs of 0.832 and 0.794, respectively.

According to the evaluation results of MSE, RMSE, precision, recall, and F1-score in Table 5, the performance of the models varies. N_XGB performs the best with an MSE of 0.0478 and an RMSE of 0.2186, both of which are the lowest, indicating that it has the most minor prediction error. The N_XGB model also performs well in terms of precision, recall and F1 score. In contrast, the RF model had an MSE of 0.2082 and an RMSE of 0.4563, both of which were the highest, indicating that the model had a significant prediction error.

**Table 5:** Comparison of model MSE, RMSE, Precision, Recall, F1 metrics.

Mothods	MSE	RMSE	Precision	Recall	F1
N_XGB	0.0478	0.2186	0.934	0.941	0.937
N_RF	0.0626	0.2502	0.912	0.926	0.918
Dn_xgb	0.1042	0.3228	0.875	0.892	0.883
Dn_RF	0.1303	0.3609	0.842	0.868	0.854
XGBoost	0.1829	0.4277	0.803	0.835	0.818
RF	0.2082	0.4563	0.761	0.806	0.782

**Fig 13:**
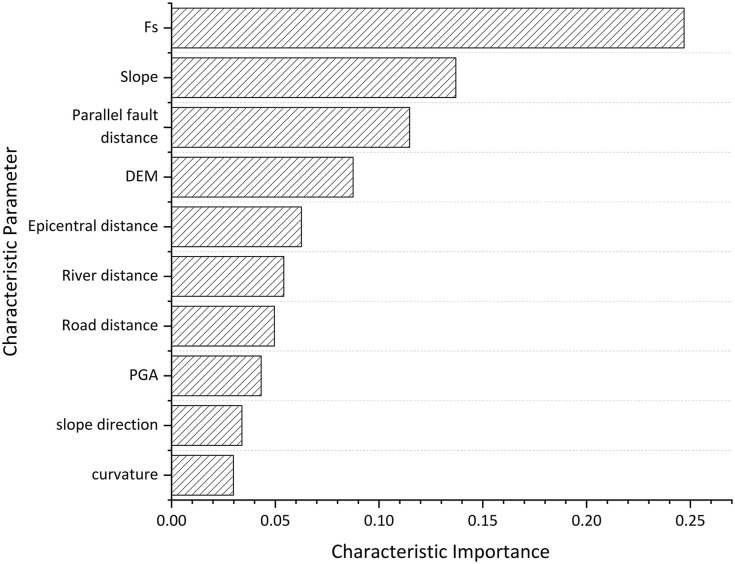
Importance ranking of feature parameters.

Upon completion of model training, the weight matrix for all nodes was extracted, and the weight values of the influencing factors were computed, as illustrated in Fig 13. This analysis revealed $F_{s}$, slope, and fault distance as critical elements affecting landslides.

PGA is acknowledged as a crucial parameter in seismic landslide research; however, its significance may differ based on the area geology and geomorphological setting. The relatively low ranking of PGA in the prediction model can be ascribed to its diminished link with landslide occurrences. Fig 2 demonstrates that the correlation between PGA and landslide distribution is not significant. Landslides are predominantly found within a PGA range of 0.18 to 0.28 g, exhibiting a somewhat homogeneous distribution. Furthermore, the majority of landslides transpire at a greater distance from the epicenter, suggesting that the influence of Peak Ground Acceleration (PGA) may be obscured by topographical elements such as slope and fault distance, which demonstrate heightened sensitivity.

**Fig 14:**
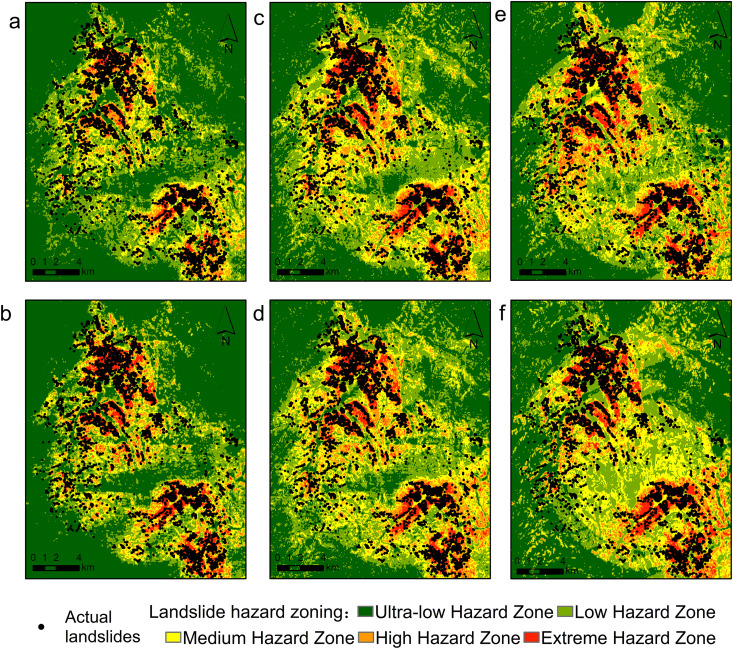
Landslide hazard zone forecast distribution map. **(a)** N_XGBoost; **(b)** N_RF; **(c)** Dn_XGBoost; **(d)** Dn_RF; **(e)** XGBoost; **(f)** RF. Source: Hazard zones modeled; regional boundaries from http://www.naturalearthdata.com/. The map was originally produced by the authors.

The trained model was applied to predict seismic landslide hazards, and the results were imported into ArcGIS. Using the natural breaks classification method, the hazard evaluation results were categorized into five zones: extremely high-risk (0.8–1), high-risk (0.6–0.8), moderate-risk (0.4–0.6), low-risk (0.2–0.4), and very low-risk (0–0.2). Fig 14 illustrates each modeled hazard zone zoning and actual landslide conditions.

To enhance the model’s predicted efficacy, the hazardous area is quantified, and the actual landslides within this perilous zone are enumerated by reclassifying the raster in ArcGIS.

**Fig 15:**
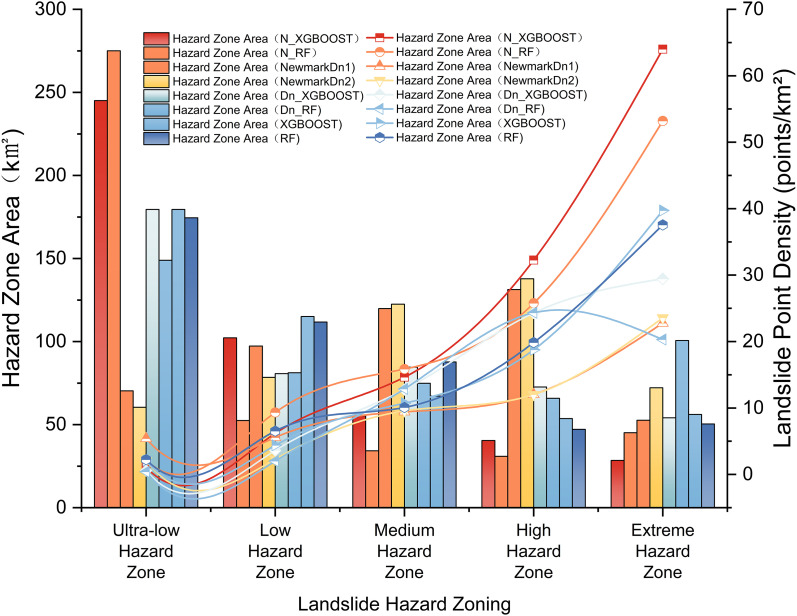
Comparison of Newmark landslide model and hybrid model results.

This study assessed six models for landslip susceptibility: Newmark integrated XGBoost (N_XGB), Newmark integrated Random Forest (N_RF), models utilizing Newmark displacements (Dn_XGB and Dn_RF), and independent XGBoost and Random Forest models. Fig 15 presents a comprehensive performance comparison across several hazard classes.

Beyond conventional performance metrics such as AUC and F1-score, it is crucial to assess whether each model accurately reflects the geomechanical mechanisms of seismic landslides and produces spatially coherent susceptibility maps. Both N_XGB and N_RF utilize the static factor of safety $F_{s}$ as a core input feature to represent pre-failure slope stability. According to the feature importance rankings shown in Fig 13, $F_{s}$ ranks highest in both models. The majority of landslide points observed in Jiuzhaigou occur on slopes with $F_{s}$ below 1.2, indicating proximity to limit equilibrium before seismic loading.

In Jiuzhaigou, slopes composed of Quaternary loess and weathered slate exhibit $F_{s}$ values of approximately 0.9 ~ 1.1. These areas are marked as high-risk by N_XGB, showing strong agreement with the mapped landslide clusters in Fig 14a. While N_RF performs similarly, its coarser tree-splitting structure occasionally produces false positives on strong lithologies—evident in Fig 14b, where certain high-risk zones do not correspond to actual landslides.

Dn_XGBoost and Dn_RF rely on cumulative Newmark displacement $D_{n}$ to characterize post-seismic slope deformation and exhibit strong detection capabilities in areas with large displacements. In Jiuzhaigou, for steep slopes and areas with high peak ground acceleration (PGA), Dn_XGBoost predicts a high landslide probability when the computed $D_{N}$ exceeds 0.2 meters. However, it tends to miss slopes that are a near failure but does not exhibit significant displacement on a saturated Permian mudstone slope in Jiuzhaigou (Fig 4), where the PGA is approximately 0.28 g, and the computed $D_{n}$ is only about 0.15 meters, the slope had an $F_{s}$ of around 1.05 close to failure and a landslide was observed in the field but not captured by the Dn-based models.

Independent XGBoost and RF models, which utilize only static features such as slope, lithology, elevation, curvature, distance to faults, epicentral distance, and PGA, are limited in their ability to differentiate between lithological strength and slope resistance. In Jiuzhaigou, slopes exceeding 35° within 10 km of the epicenter were broadly classified as high-risk by XGBoost, resulting in an excessive false positive rate. RF exhibited similar spatial bias with more diffuse high-risk zones (Fig 14e, 14f). These models lack the geotechnical foundation necessary to interpret slope stability and seismic response accurately.

Quantitative spatial analysis confirms these patterns. As shown in Fig 15, N_XGB achieved the best balance between landslide coverage and spatial precision, predicting 5.61 km^2^ of landslides in very high-risk zones and achieving a landslide point density of 64.02 points/km^2^. N_RF followed with 6.59 km^2^ and 53.22 points/km^2^. Dn_XGBoost and Dn_RF predicted 4.84 and 5.15 km^2^, respectively, but had lower point densities (39.72 and 37.56 points/km^2^). In contrast, standalone XGBoost and RF predicted smaller landslide areas (3.36 and 4.63 km^2^), yet overestimated total hazardous areas to 54.12 km^2^ and 100.63 km^2^, leading to diluted susceptibility maps and poor hazard zoning resolution.

Additionally, N_XGB maintained high landslide density within very hazardous zones while minimizing overprediction in low-risk areas, offering a well-balanced delineation strategy suitable for practical mitigation. $D_{n}$-based models, in contrast, displayed both conservative and aggressive tendencies across different regions, reflecting their sensitivity to $D_{n}$ thresholds. These tendencies limit their adaptability across diverse geological conditions. The standalone XGBoost and RF models not only lacked precision but also failed to capture clustered landslide distributions—critical for focused risk management.

Overall, N_XGB aligns best with geological observations. It identifies slopes with $F_{s}$ < 1.2 and PGA exceeding seismic thresholds, embodying the dual triggering mechanism of static instability and dynamic disturbance. N_RF performs second best but tends to overgeneralize. $D_{n}$-based models prioritize large-displacement areas but miss low $D_{n}$, near-failure slopes. The independent models rely heavily on slope and seismic intensity, lacking geomechanical context. The ranking by geological consistency matches the AUC hierarchy: N_XGB > N_RF > Dn_XGBoost > Dn_RF > XGBoost > RF. These results demonstrate that only models explicitly integrating physical-process indicators can simultaneously ensure high predictive accuracy and interpretability, making them suitable for both scientific understanding and hazard management.

Compared with similar studies in Table 6, this study highlights the advantages of the proposed method by comparing the characteristic parameters and prediction accuracies of existing seismic landslide susceptibility models. The logistic regression model of Ma et al. [48] and the evidence-weighted method of Yang et al. [49] achieve prediction accuracies of 0.89 and 0.87, respectively, by considering factors such as slope gradient, slope direction, lithology, and distance from the fault. Zhang et al. [50] improved the prediction accuracy to 0.90 by using the BP neural network but did not fully consider the multidimensional factors affecting earthquake-induced landslides. Wang et al. [51] proposed a Transformer model, which incorporated topographic, geologic, and climatic factors, but the prediction accuracy was lower, only 0.87. In contrast, the model proposed in this paper combines the Newmark model with machine learning techniques (N_XGBoost) to incorporate a broader set of feature selections. By incorporating parameters such as slope, face width, fault distance, peak ground acceleration (PGA), and curvature, the model achieves a prediction accuracy of 0.96, which significantly improves accuracy and robustness.

**Table 6:** Comparison of results of similar studies.

Similar studies	Ma et al.	Yang et al.	Zhang et al.	Wang et al.	This Study
Model	LR	Evidence Weighting Method.	BP Neural Network	Transformer Model	N_XGBoost N_RF
Characteristic Parameter	Slope, elevation, slope direction, lithology, vertical fault distance, horizontal fault distance, epicenter distance, river distance, road distance, TPI index	Slope, slope direction, elevation, lithology, fault distance, PGA, river distance	Slope, slope direction, elevation, lithology, vertical fault distance, horizontal fault distance, epicenter distance, river distance, road distance, TPI index	elevation, slope, TWI, topographic relief, lithology, faults distance, Seismic intensity, annual rainfall, water distance, roads distance, land use, slope direction.	Fs, slope, slope direction, elevation, curvature, fault distance, PGA, river distance, road distance, epicenter distance
Predictive Accuracy	0.89	0.87	0.90	0.87	0.96

### Generalization capabilities of Newmark-based machine learning models

**Fig 16:**
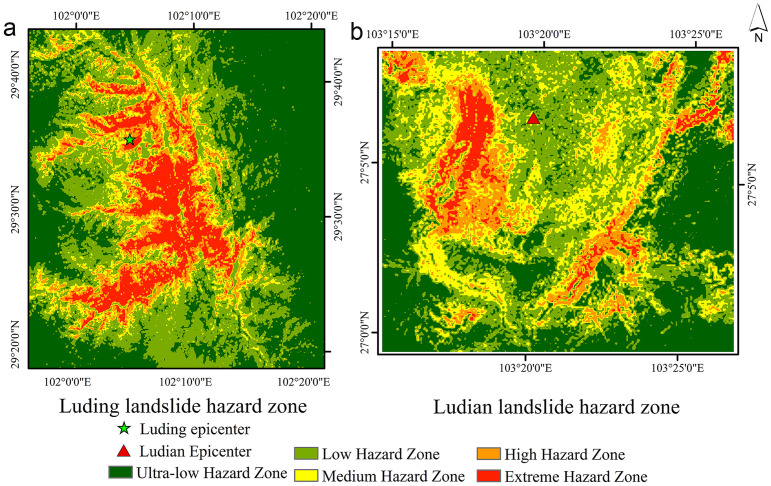
Validation area. **(a)** Luding; **(b)** Ludian landslide hazard zones. Source: Hazard zones modeled; regional boundaries from http://www.naturalearthdata.com/. The map was originally produced by the authors.

To test model transferability beyond Jiuzhaigou, we applied N_XGB to two contrasting regions: Ludian and Luding. Ludian’s 2014 Ms 6.5 earthquake produced PGAs of 0.38–0.42 g—well above Jiuzhaigou’s 0.28 g—and triggered 1 024 landslides clustered 4–8 km from the fault and adjacent to roads and rivers. In this setting, N_XGB achieved an AUC of 0.88, accurately delineating extremely high- and high-risk zones while avoiding overprediction in lower-risk areas. Luding, by contrast, features mid-elevation mudstone slopes (1300–1700 m), gentler slopes (25°–35°), and PGAs of 0.36–0.44 g, yet still yielded an AUC of 0.86 when mapped with N_XGB. Despite differences in seismic intensity, elevation band, slope class, and substrate, N_XGB consistently identified clustered landslide zones and minimized false alarms, demonstrating strong generalizability across diverse earthquake-triggered landslide environments. Fig 16 shows the landslide hazard map of the Ludian area and the Luding area.

The models effectively identified high-risk areas while maintaining accuracy in moderate- and low-risk zones. This approach facilitates large-scale identification of landslide risk, especially at very high and high hazard levels. The models also identified potential landslide points with high density, showing notable accuracy in densely clustered areas. In low-risk zones, the models reduce false alarms despite missing some landslide points, improving the overall prediction reliability. These results indicate that Newmark-based machine learning models are highly applicable and generalizable for earthquake-induced landslide prediction.

## Discussion

This work presents a hybrid methodology that combines machine learning techniques with the Newmark model to improve landslip susceptibility evaluation. The model integrates data-driven methodologies with physical principles, accounting for static and dynamic elements, including geography, hydrology, seismic activity, and anthropogenic effects, to provide thorough risk assessments. Critically, this approach addresses key limitations observed in existing models. Purely data-driven methods often fail to incorporate seismic dynamic parameters, such as PGA, and physical mechanisms, as evidenced by the significant drop in generalization accuracy to 76% in Karakas et al. [27] random forest model for the 2023 Türkiye double earthquakes. Similarly, statistical models exhibit weak physical interpretability due to neglected mechanical coupling; for instance, Matsakou et al. [28] LSI/LR model for Lefkada earthquakes misjudged high-risk slope angles as 60°–80° while field observations confirmed actual concentrations at 40°–50°.

Zeng et al. [52] presented a physical-environmental coupling model (PECM) that incorporates Newmark displacement (*Dn*) alongside characteristics of slope, fault distance, hydrology, and human activity. Their N-SVM model attained a good prediction accuracy (AUC = 0.937). Nonetheless, dependence on GIS-based buffer sampling and empirically obtained *Dn* values constrained its applicability across many geological contexts. This study utilizes a physics-informed sampling strategy grounded in Newmark model partitioning, which differs fundamentally from traditional GIS buffer sampling approaches. While buffer-based methods often rely on fixed-distance zones around faults or epicenters—potentially introducing subjective or arbitrary boundaries—the proposed strategy leverages physical hazard zoning results to inform sampling directly. This ensures that non-landslide samples are selected from zones objectively identified as low-hazard based on geomechanical simulation outcomes, thereby enhancing the scientific robustness and representativeness of the training dataset. Strengthening generalisability, minimizing sampling subjectivity, and improving the model’s capacity to differentiate between landslide and non-landslide areas.

Li et al. [53] emphasized the advantages of employing *Dn* for enhanced spatial representation and feature extraction in landslide vulnerability evaluations. Although their methodology enhanced predicted accuracy, they recognized the constraints of empirically obtained *Dn* parameters, necessitating additional validation across various geological contexts. This study utilizes Monte Carlo simulations (MCS) to handle parameter uncertainty, offering a more comprehensive characterization of geotechnical parameters, including friction angle and cohesiveness. This improvement allows the hybrid model to represent essential physical features precisely across diverse seismic events.

To address the limitations inherent in the rigid-block assumption of the Newmark model, this study adopts several targeted strategies to reflect real-world geomechanical behavior better. First, Monte Carlo Simulation (MCS) incorporates the variability and uncertainty of key geotechnical parameters (e.g., cohesion, friction angle, unit weight), accounting for spatial heterogeneity in subsurface conditions. Second, instead of directly relying on deterministic Newmark outputs, we introduce the calculated static factor of safety ($F_{s}$) and displacement ($D_{n}$) into machine learning models as physical-informed features. These features capture both the static and dynamic response of the slope under seismic loading, enabling the model to internalize complex physical behaviors beyond the capacity of the simplified Newmark framework. Furthermore, integrating Mw in the multiparameter Newmark displacement equation indirectly accounts for regional seismic amplification effects. Combined with the data-driven capability of XGBoost to capture non-linear interactions among topography, lithology, and seismic intensity, this hybrid approach substantially mitigates the oversimplifications of the classical rigid-block mechanism. Consequently, the proposed framework acknowledges and proactively addresses the mechanical and dynamic complexities often neglected in traditional Newmark analyses.

This study analyzes six landslide susceptibility models in order to provide a thorough evaluation of the model. These models include standalone XGBoost and RF models, models that incorporate *Dn* as a feature (Dn_XGB and Dn_RF), and combinations of the Newmark model with XGBoost (N_XGB) and Random Forest (N_RF). According to the results, the N_XGB model achieves an AUC of 0.96 and offers more precise and dependable spatial forecasts, while solo models perform poorly, especially in high-risk locations. Interestingly, models that included *Dn* (Dn_XGB and Dn_RF) performed worse than models based on (N_XGB and N_RF), even though *Dn* was added as a feature to improve predictive capability.

The inherent distinctions between $F_{s}$* * and *Dn* are the reason for the Newmark-based models’ better performance. Uses proven physical principles to directly integrate important hydrological and geotechnical factors, including lithology, water content, and slope stability. However, when employed as an independent feature, *Dn*, which is built from empirical approximations, can provide a less explicit representation of these aspects, which could reduce its predictive effectiveness. Additionally, the sample selection procedure based on Newmark model findings naturally incorporates the influence of *Dn* into the N_XGB and N_RF models. Consequently, its influence is mirrored in the model architecture, improving predicted accuracy without the need for extra input, even if *Dn* is not explicitly included as a standalone feature.

In practical terms, the proposed hybrid model offers substantial value for real-world disaster management and infrastructure planning. The high-resolution hazard zoning outputs can support post-earthquake emergency response by rapidly identifying areas with the highest landslide risk and prioritizing rescue and resource allocation. For infrastructure development, the predicted hazard zones can inform safer siting of roads, power lines, and public facilities, particularly in mountainous seismic zones. Moreover, the model’s quantitative outputs can be directly integrated into regional land-use planning and policy formulation, enabling authorities to delineate restricted construction zones and establish early-warning thresholds grounded in geophysical and empirical evidence.

The model maintains strong adaptability regarding regions with limited data or computational capacity. Most required input parameters—such as DEM, geological formations, fault lines, and seismic intensity—are obtainable from open-source national or international geospatial databases. The machine learning components (XGBoost and RF) are lightweight regarding computational demand, and training can be completed on standard desktop environments. The model can also be deployed in low-resource settings using pre-trained weights from similar geotectonic regions, followed by minimal local calibration. Furthermore, even without ML components, the Newmark module provides rapid, interpretable hazard zoning, serving as an accessible first-tier assessment method for emergency preparedness in data-constrained environments.

There are still difficulties despite these developments. The intricate deformation and failure mechanisms of actual landslides are oversimplified by the Newmark model’s dependence on strict block assumptions. Further challenges are presented by the hybrid framework’s computing requirements and the scarcity of high-resolution hydrological and geotechnical data. In order to overcome these constraints, future research should incorporate more realistic deformation models and sophisticated geomechanical simulations, improve parameter estimation using data fusion and remote sensing methods, and include real-time data streams to improve responsiveness during disaster events. The model’s usefulness might be enhanced by considering long-term climate influences and seasonal hydrological fluctuations.

The hybrid model significantly advances landslide prediction and mitigation efforts by providing a scalable solution for risk management, early warning systems, and real-time catastrophe response in various seismic zones.

## Conclusion

This study used machine learning techniques and the Newmark physical landslide displacement model to construct N_XGBoost models for earthquake-induced landslide prediction. The following deductions were made:

[1] The multiparameter prediction equation that uses both PGA and Mw (Dn2) performs better in the Newmark model than the single-parameter equation that uses only PGA (Dn1). However, the N_XGBoost model performs noticeably better than the physical model, combining physical concepts with machine learning techniques.
[2] Nonlandslide locations were sampled using hazard evaluations produced by the Newmark model, and earthquake-induced landslide data from the Jiuzhaigou region was used for model training and testing. N_XGBoost, N_RF, Dn_XGBoost, Dn_RF, standalone XGBoost and standalone RF models were among the six landslide susceptibility models compared. The results showed that standalone models performed significantly worse. The N_XGBoost model had the highest accuracy, with an AUC of 0.96 and an MSE of 0.0478, outperforming other models across all hazard levels. For identifying landslide-prone locations and lowering the possibility of needless interventions, $F_{s}$ proved to be a more thorough and dependable feature than the modest gains made by adding Newmark displacement ($D_{n}$), demonstrating that pre-failure stability captures key mechanics missed by displacement‐only approaches. Slope gradient, $F_{s}$, and proximity to active faults emerged as the dominant controls on landslide occurrence, modulated by rock mass properties, rivers, and roads; lower landslide risk is linked to more complicated rock layers. Steep slopes and locations near faults, rivers, and roadways are more likely to have landslides.
[3] When validated in Ludian (2014 earthquake, PGA 0.38–0.42 g) and Luding (PGA 0.36–0.44 g) regions, N_XGB maintained strong performance (AUC = 0.88 and 0.86, respectively), accurately identifying high-density slide clusters and minimizing false alarms despite differing elevation bands, slope classes, and lithologies. This confirms the framework’s generality for regional-scale hazard assessments.
[4] Although the physics–data hybrid models demonstrate excellent accuracy and regional generalizability, there remains room for improvement. First, advanced deep learning architectures such as graph neural networks or temporal Transformers could be incorporated to better capture higher-order spatiotemporal dependencies in topographic, geological, and seismic data. Second, multisource dynamic inputs—such as multitemporal InSAR deformation, radar-based precipitation, soil moisture, and vegetation indices—should be integrated to enable continuous, end-to-end monitoring and modeling of the entire landslide lifecycle. Furthermore, real-time seismic and rainfall data should be dynamically incorporated into model updates and rapidly adapted to diverse tectonic and climatic contexts, thereby establishing a truly real-time, cross-regional landslide early warning system.
